# Invasive infections by group A Streptococcus infections in Brazil: a pediatric case series

**DOI:** 10.1590/1984-0462/2025/43/2024100

**Published:** 2024-12-20

**Authors:** Daniela Otoni Russo, Marina Melo Moreira, Aline Almeida Bentes, Bárbara Araujo Marques, Ericka Viana Machado Carellos, Flávia Duarte Nogueira, Roberta Maia de Castro Romanelli, Ana Luisa Lodi Jimenez, Carolina Campos Vieira de Souza, Luana Casilho Moreira, Milena Pereira Parreira, Renata Araujo Avendanha, Renata Campos Correa dos Santos, Márcio Antônio Ferreira Arantes, Lilian Martins Oliveira Diniz

**Affiliations:** a Fundação Hospitalar do Estado de Minas Gerais, Hospital Infantil João Paulo II, Belo Horizonte, MG, Brazil.; b Universidade Federal de Minas Gerais, Belo Horizonte, MG, Brazil.; c Faculdade Ciências Médicas de Minas Gerais, Belo Horizonte, MG, Brazil.

**Keywords:** Streptococcus pyogenes, Group A streptococcal infection, Pediatrics, Infectious diseases, Streptococcus pyogenes, Infecção estreptocócica do Grupo A, Pediatria, Doenças infecciosas

## Abstract

**Objective:**

Group A Streptococcus (GAS) are Gram-positive cocci that colonize the nasopharynx and/or skin and in rare cases may cause severe invasive infections. Although these infections decreased during the COVID-19 pandemic, some countries have observed an increased number of invasive GAS (iGAS) diseases in recent years. The objective of this study was to describe a series of iGAS diseases in a referral hospital for the treatment of pediatric infectious disease in Minas Gerais State, Brazil, between September 2022 and August 2023. The data were obtained from medical records, and GAS was isolated from blood, pleural, cerebrospinal, or joint fluid.

**Case description:**

A total of nine patients, four male and five female, between eight months and ten years of age, were admitted. Of the patients observed, three (33%) had pneumonia, three (33%) had toxic shock syndrome, three (33%) had septic arthritis, one (11%) had osteomyelitis, two (22%) had meningitis, and two (22%) cellulitis. The majority of patients (5) experienced complications, including pleural effusion, septic shock, cerebral vasculitis, and limb amputations. In total, seven (75%) patients were admitted to the intensive care unit, and one patient died.

**Comments:**

It is important to be aware of the possibility of *S. pyogenes* infection in children with pulmonary, neurological, osteoarticular, skin infections or in severe cases of septic shock. Given the absence of specific clinical signs, epidemiological data showing an increase in the local incidence of *S. pyogenes* infections is of great importance and should serve as an alert for the diagnosis of the disease.

## RESUMO

**Objetivo:**

Streptococcus do grupo A (SGA) são cocos gram-positivos que colonizam a nasofaringe e/ou a pele e em casos raros podem causar infecções graves. Embora essas infecções tenham diminuído durante a pandemia da COVID-19, alguns países observaram um aumento no número de doenças invasivas pelo SGA nos últimos anos. O objetivo deste estudo foi descrever uma série de doenças invasivas pelo *S. pyogenes* em crianças internadas em um hospital de referência para tratamento de doenças infecciosas em Minas Gerais, Brasil, entre setembro de 2022 e agosto de 2023. Os dados foram obtidos dos prontuários médicos. O SGA foi isolado do sangue, líquido pleural, cerebrospinal ou fluido articular.

**Descrição do caso:**

O total de nove pacientes, quatro do sexo masculino e cinco do sexo feminino, com idade entre oito meses e dez anos, foram admitidos. Ao todo, três (33%) tiveram pneumonia, três (33%) síndrome do choque tóxico, três (33%) artrite séptica, um (11%) osteomielite, dois (22%) meningite e dois (22%) celulite. A maioria dos pacientes (5) apresentou complicações, incluindo derrame pleural, choque séptico, vasculite cerebral e amputações de membros. Ao todo, sete (75%) pacientes foram admitidos na unidade de terapia intensiva, e um paciente morreu.

**Comentários:**

É importante estar atento à possibilidade de infeção por *S. pyogenes* em crianças com infecções pulmonares, neurológicas, osteoarticulares, cutâneas ou em casos graves de choque séptico. Diante da ausência de sinais clínicos específicos, dados epidemiológicos que demonstrem o aumento da incidência da doença são de grande importância e devem servir de alerta para o diagnóstico da doença.

## INTRODUCTION

Group A Streptococcus (GAS), also known as *Streptococcus pyogenes*, is a Gram-positive coccus transmitted through close contact with an infected person, mainly through respiratory secretions or wounds.^1,2^ The infection usually causes mild illnesses such as tonsillitis, pharyngitis, impetigo, cellulitis and scarlet fever, being the most common cause of bacterial pharyngitis in school-aged children, with an incidence peak in winter and spring months.^3^ Additionally, GAS can lead to non-suppurative complications like rheumatic fever and post-streptococcal interstitial nephritis. In rare cases, GAS may cause severe and life-threatening infections known as invasive Group A Streptococcal diseases (iGAS), that manifest as bacteremia, pneumonia, meningitis, toxic shock syndrome or necrotizing fasciitis, and can also involve bones and joints.^1,3^

During the COVID-19 pandemic, there was a 25% decrease in infections caused by GAS such as pharyngitis and scarlet fever, across all age groups.^1^ This decrease was likely due to measures adopted to prevent the spread of respiratory diseases, such as the closure of schools and workplaces, as well as the widespread use of masks and the practice of physical distancing.^1^ Following the release of isolation measures, some countries worldwide observed a rise in GAS infections.^3^ In December 2022, the World Health Organization issued an alert regarding a significant increase in cases of iGAS in children aged 0–10 years in various European countries.^3,4^ Several countries reported deaths associated with iGAS in children under ten years old during the same period.^3^

In Brazil, between 2018 and 2023, there were a total of 784 requests for tests for iGAS infections, with a decrease in the number of requests from 2020 onwards and a new increase from June 2023. In February 2024, the Ministry of Health issued a warning note reporting an increase in the number of cases in some states across the country, in order to guide health professionals in the laboratory identification of invasive infections caused by these bacteria, as an attempt to determine the precise number of cases.^5^

In August 2023, the Health Department of Minas Gerais State, Brazil, issued an alert reporting the death of a child due to shock caused by *S. pyogenes*.^6^ A survey of invasive *S. pyogenes* infections at João Paulo II Children’s Hospital, the main public hospital for the treatment of pediatric infectious diseases in Belo Horizonte, Minas Gerais state capital, was performed from 2018 to 2023. We identified one patient hospitalized in 2018, two in 2019, one in 2020, none in 2021 and one in 2022. In 2023, an overall increase in the number of cases was identified, resulting in eight patients admitted. Considering the epidemiological data, in this study we aimed to describe the patients hospitalized at Hospital Infantil João Paulo II throughout 2022 and 2023, who presented with iGAS disease. This study was approved by the hospital’s ethics committee (Certificate of Presentation for Ethical Appreciation — CAAE no. 78366024.4.0000.5119).

## CASE REPORT

From July 2022 to September 2023, all children aged 0–13 years that were hospitalized with iGAS infections at João Paulo II Children’s Hospital, in Belo Horizonte, were studied. Invasive infection was defined as laboratory isolation of GAS from any sterile site in a patient with pneumonia, toxic shock syndrome, osteoarticular infections, meningitis or cellulitis. The hospital’s laboratory follows the standardization of the Brazilian Committee for Antimicrobial Sensitivity Testing (BrCAST). After isolating and identifying the microorganism, *Streptococcus pyogenes* cultures are sown on Mueller-Hinton Agar culture medium for the disc diffusion test to assess the bacteria’s sensitivity to penicillin, cephalosporin and carbapenem. Phenotypic resistance tests are carried out at the local hospital, and strains are sent to the state referral laboratory, Fundação Ezequiel Dias (FUNED), for genotypic testing.

During the study period, nine patients with iGAS infections were admitted to João Paulo II Children’s Hospital, comprising four males and five females aged between eight months and ten years. Among the nine patients, two (22%) were less than one year old, four (44%) were between one and five years old, and three (33%) were older than five. The iGAS infections included pneumonia (3), toxic shock syndrome (3), septic arthritis (3), osteomyelitis (1), meningitis (2), and cellulitis (2) (Table 1). More than one diagnosis was present in over half of the patients upon admission. The bacteria were isolated from blood cultures (4), pleural fluid cultures (3), joint fluid cultures (2), and cerebrospinal fluid cultures (2). In one patient, blood and cerebrospinal fluid cultures were obtained postmortem. All the isolates were sensitive to penicillin, clindamycin, erythromycin, levofloxacin, rifampicin and sulphamethoxazol/trimetoprim.

**Table 1:** Clinical characteristics and treatment of 9 children presenting with invasive *S. pyogenes* infection.

Patient	Age	Clinical manifestations	Treatment	Total length of treatment	Diagnosis
1	8 months	Fever, rash, petechiae, irritability, weak pulses, cyanosis, mottled skin, tachypnea, cough, respiratory distress	Ceftriaxone, oxacillin, clindamycin. Transfusion of red blood cells, plasma and platelets Pleural drainage	15 days	Pneumonia Toxic shock syndrome
2	5 years 2 months	Fever, prostration, edema in the left upper and right lower limb with limited movement	Ceftriaxone, Penicillin G, clindamycin, oxacillin, ampicillin	15 days	Toxic shock syndrome Cellulitis
3	4 years 11 months	Pain and swelling of the right heel and left lower limb	Ceftriaxone, penicillin G, amoxicillin	21 days (7 in hospital, 14 at home)	Acute osteomyelitis on the right heel Cellulitis on the left leg
4	9 years	Fever, pain, and swelling in the right ankle	Ceftriaxone, oxacillin, amoxicillin Arthrotomy	21 days	Septic arthritis
5	3 years 5 months	Fever, pain, and swelling in the right knee, limited ambulation, edema and hyperemia in the left ankle	Oxacillin, cephazolin, penicillin Arthrotomy	20 days	Septic arthritis Cellulitis
6	8 months	Fever, pain, and swelling in the right knee, limited ambulation, edema and hyperemia in the left ankle	Ceftriaxone Clindamycin, amoxicillin Pleural drainage	11 days	Pneumonia
7	3 years	Fever, chest pain, tachypnea, petechial rash	Ceftriaxone Ampicillin, clindamycin, oxacillin Pleural drainage	12 days	Pneumonia
8	10 years	Fever, headache, neck pain, neck stiffness	Ceftriaxone	14 days	Meningitis
9	1 year 5 months	Fever, cyanosis, petechial rash	-	-	Meningitis Toxic shock syndrome Septic arthritis (?)

Upon admission, none of the patients had pre-existing conditions. All patients presented fever as one of their initial symptoms. Respiratory distress was observed in three patients diagnosed with pneumonia. One patient diagnosed with meningitis showed signs of meningeal irritation, and three patients diagnosed with toxic shock syndrome had signs of hemodynamic instability. Five patients presented impaired limb movement due to swelling and pain of joints, arms and legs, and were diagnosed with arthritis, osteomyelitis, or cellulitis. One patient was admitted in shock and cardiac arrest and died shortly after admission. The child was one year and five months old and had a previous history of fever for six days. The day before she died, she had been taken to hospital with pain in her left ankle. She was assessed by the orthopedic department and had an X-ray of the limb, without alterations. The following day, according to the parents, the child was more prostrate, had lip cyanosis and a rash on lower limbs. She was then taken again to the hospital but was admitted in cardiac arrest and underwent unsuccessful cardiac resuscitation maneuvers. After death, due to suspected meningococcal disease, a cerebrospinal fluid exam (CSF) was performed which revealed protein=102 mg/dL, glucose=44 mg/dL, white blood cell count (WBC)=80 cells/mm^3^ (lymphocytes 56%, monocytes 28%, neutrophils 16%) and red blood cells count=8,400 cells/mm^3^. *S. pyogenes* was found in patient’s blood and CSF. Despite that, no neurological symptoms were reported (Table 1).

In total, seven patients were initially treated with third-generation cephalosporin and one received crystalline penicillin. Additionally, five patients were also given oxacillin, cefazolin or clindamycin for suspected staphylococcal infection and two patients also received clindamycin for the treatment of toxic shock syndrome (Table 1). For most patients, treatment was switched to penicillin G when *S. pyogenes* was identified, except the patient diagnosed with meningitis who was treated only with ceftriaxone. The duration of treatment ranged from 11 to 74 days and two of them were sent home receiving oral antibiotics. Among the patients who presented with toxic shock syndrome, two progressed with improvement of the shock within 48–72 hours after starting the antibiotic. By the time *S. pyogenes* was identified, they had all come out of the most severe phase, which is why immunoglobulin was not used. Unfortunately, the third patient with streptococcal toxic shock died so quickly that it was not possible to start antibiotics or immunoglobulin. In addition to treatment with antibiotics, three patients with pneumonia underwent thoracic drainage for pleural effusion, two out of the three patients with arthritis had joint drainage, and the patient diagnosed with osteomyelitis had surgical debridement of the affected bone.

Out of the eight patients who remained hospitalized, six experienced complications during their stay. Three patients with pneumonia developed pleural effusion, and two of them also progressed to septic shock. One patient with meningitis had cerebral vasculitis and respiratory failure, and another patient with septic shock developed disseminated intravascular coagulation with lower limb and finger amputations. A patient with clinical diagnosis of acute osteomyelitis on the right foot was initially treated with ceftriaxone followed by six days of intravenous penicillin G, discharged with an additional 14 days of oral amoxicillin. Despite oral antibiotics, she was readmitted after 11 days with fever, pain and swelling of the left limb. A plain radiograph revealed osteomyelitis of the left tibia and she was started on intravenous penicillin G for more three weeks. Due to the recurrence of symptoms despite previous treatment with antibiotics, a diagnosis of chronic osteomyelitis was made, and a surgical debridement of the lesion was also carried out. After this period, the child was sent home and was given oral amoxicillin for more than three weeks, with a good recovery (Table 2).

**Table 2:** Complications and outcomes of 9 children presenting with invasive *S. pyogenes* infection.

Patient	Complications observed during hospitalization	Length of hospital stay	Culture (material)	Intensive Care Unit	Outcome
1	Pleural effusion Acute renal failure Amputation of lower limbs and fingers of the hands	74 days	Pleural fluid	Yes	Discharged
2		18 days	Blood	Yes	Discharged Despite the improvement, the child still presented limited mobility of the right lower limb due to the cellulitis associated with prolonged hospitalization
3		11 days	Blood	Yes	Discharged on oral amoxicillin for more 14 days The patient was readmitted after 11 days due to fever and pain on the left leg. She was diagnosed with chronic osteomyelitis of the left tibia, had a surgical debridement and was treated for another 6 weeks.
4		10 days	Blood and joint fluid	No	Discharged
5	Shock	21 days	Joint fluid	No	Discharged
6	Pleural effusion Shock	11 days	Pleural fluid	Yes	Discharged
7	Pleural effusion Shock	14 days	Pleural fluid	Yes	Discharged
8	Respiratory failure Cerebral ischemia (Seizure, hemiparesis, aphasia)	22 days	Cerebrospinal fluid	Yes	Discharged
9	Respiratory failure Cardiac arrest	1 day	Blood and cerebrospinal fluid	No	Death

Among the nine patients hospitalized, six required intensive care during their stay. At the end of the study period, eight children had been discharged, one of them after a second hospitalization and one child had died (Table 2).

## DISCUSSION

The prevalence of *S. pyogenes* infections in children is uncertain and the general understanding of the disease caused by SGA remains relatively poor in comparison to other infectious diseases. Many countries with established infectious disease surveillance programs undertake relatively little surveillance of diseases caused by *S. pyogenes* and other pyogenic streptococci. However, in 2022, France, Ireland, Sweden and the United Kingdom observed an increase in iGAS infections, which mainly affected children under ten years of age.^4^ A survey conducted in the Netherlands revealed that in 2022 there were 61 recorded pediatric cases, compared to 56 cases observed between 2018 and 2019.^4^ Early serotyping data suggested that the rise in GAS cases in children was not related to a new strain or an increase in antimicrobial resistance. Instead, data have shown that it was related to a greater circulation of respiratory viruses, including the influenza virus and the respiratory syncytial virus, showing that co-infection could increase the risk of GAS disease.^4^ Enhanced surveillance measures were implemented in countries where an increase in invasive cases was reported, along with public health messages aimed at the general population and clinicians, in order to improve detection and management, with early recognition, notification, and immediate initiation of treatment throughout 2022 and 2023.^3^

Invasive GAS infections are responsible for a diverse range of clinical manifestations. A study conducted in Spain found that 15.4% of invasive infections caused by *S. pyogenes* occurred in the skin and soft tissues, manifesting as cellulitis or necrotizing fasciitis. Breakdown of the protective barrier and skin wounds can precede and precipitate soft tissue infection by GAS.^6^ The three patients in our study who developed cellulitis also presented associated conditions such as osteomyelitis, septic arthritis and septic shock. Data from literature show that cellulitis can be associated with other iGAS infections, as we could observe.^7^ When skin lesions are disseminated and accompanied by systemic toxicity, necrotizing fasciitis should be considered, and local debridement should be evaluated.^8^ In this study, all patients with skin infections received treatment with antibiotics only, and had no signs of necrotizing fasciitis.

Pneumonia is a severe manifestation of iGAS and is reported as an uncommon cause of acute respiratory infections in children. It generally occurs after viral episodes and is associated with a higher risk of mortality.^9-12^ The onset of the condition is generally abrupt and the disease is characterized by chills, fever, dyspnea, chest pain and cyanosis.^11,12^ The three patients described here presented complicated conditions with pleural effusion and shock, which is consistent with literature data indicating high rates of complications.^11,12^ Shock is reported in around 10–15% of cases, and other complications such as necrotizing pneumonia, mediastinitis, pericarditis, pneumothorax, and bronchiectasis may also occur.^10,11,13^ The disease typically has a long clinical course and is associated with prolonged hospitalization.^11,12,14,15^

Infections of the central nervous system (CNS) caused by GAS remain rare, accounting for less than 0.2% of all bacterial meningitis.^16^ A review of literature between 1996–2006 identified only 13 cases, two reported in Brazil.^17^ The clinical presentation of the disease is similar to other bacterial meningitis and may include fever, neck stiffness, vomiting, and headache. The pathogenesis of primary GAS meningitis is unclear. Any interventional procedure in patients with otitis media, pharyngitis, or head trauma may predispose them to CNS infection.^18^ In our study, no predictive factors were observed to justify meningeal involvement of *S. pyogenes*. One of the children presented severe sepsis which probably preceded the infection in the CNS through hematogenous dissemination. The diagnosis of infection in the CNS was made only after death. Progression to neurological complications and sequelae is common, with a fatality rate that varies between 12 and 26%.^16^

Septic arthritis caused by GAS is considered an uncommon event. However, it should not be overlooked. A French cohort, carried out in 2020, evaluated 337 children with joint infections, and *S. pyogenes* was identified in 15% of them.^19^ The microorganism can penetrate the joint space through hematogenous dissemination, contiguity due to open fractures and traumatic injuries or direct inoculation after orthopedic procedures.^19,20^ In our series, the three patients with septic arthritis presented the hematogenous route as the probable mechanism of infection and showed classic symptoms with monoarticular involvement with pain, edema and movement impairment.^21^ The treatment of choice is antibiotic therapy associated with local drainage.^20^

Bone GAS infection can also occur, and has been associated with arthritis in 21 to 42% of cases.^20^ The coexistence of osteomyelitis and septic arthritis has been associated with older age, longer duration of symptoms, bacteremia and longer hospital stay.^22,23^ The treatment of osteomyelitis is based on antibiotic therapy and in some situations such as abscess, bone sequestration or therapeutic failure, a surgical approach is recommended, as we could observe in our patient.^22^ In this case, conservative treatment had an unfavorable outcome, requiring bone debridement.

Streptococcal toxic shock (STSS) is the most severe manifestation of the invasive diseases caused by GAS and is characterized by hypotension and multisystem involvement.^12,24^ STSS may occur with infection at any site but most often occurs in association with infection of a cutaneous lesion. Signs of toxicity and a rapidly progressive clinical course are characteristic, and the case fatality rate may exceed 50%. The illness is defined by the following clinical manifestations: hypotension associated with multi-organ involvement characterized by two or more of the following: renal impairment, coagulopathy, liver involvement, acute respiratory distress syndrome, generalized erythematous macular rash that may desquamate and soft-tissue necrosis. A patient that meets the clinical case definition associated with isolation of *S. pyogenes* from a normally sterile site (e.g., blood or cerebrospinal fluid or, less commonly, joint, pleural, or pericardial fluid) is defined as a confirmed case. The release of cytokines induced by bacterial toxins causes loss of fluid from the intravascular compartment, resulting in STSS symptoms.^12,24^ In a retrospective study conducted in Texas fever was the most common symptom, followed by non-specific gastrointestinal symptoms and respiratory discomfort. Other infections caused by GAS can generally be complicated by STSS in about 30% of cases.^24^ Risk factors for disease progression to STSS are not yet well defined.^25^ In this series of cases, three patients presented STSS associated with other possible diagnoses. The prognosis of STSS is poor, as we could see in our patients, with reported deaths in 30 to 60% of patients.^12,24^ In our study, only one patient had a favorable outcome, one child required limb and finger amputations, and another child died shortly after hospital admission.

Penicillin is the preferred treatment for confirmed cases of iGAS.^8^ Strains that are resistant to penicillin are rare and, if detected, should be genotyped to assess the presence of genetic mutations.^25^ None of the strains identified in this study were found to be resistant to penicillin. For all severe cases of iGAS, it is highly recommended to add clindamycin, a potent inhibitor of toxin production, as it has been associated with improved outcomes.^9^ Despite antibiotics, the use of intravenous immunoglobulins plays a vital role in neutralizing cytokines that can cause capillary leak, hypotension, and circulatory failure. In some cases, other specific treatments such as surgical debridement of necrotic tissue and surgical drainage of empyema might also be necessary.^25^

The process of human infection by GAS is complex and multifactorial, involving both host and bacterial factors that contribute to the pathogenesis of the disease. As was observed in other countries, Brazil has also faced a challenge with the rise of iGAS infections in children. Despite the rarity of serious manifestations associated with SGA, it is important for health professionals to be aware of the possibility of *S. pyogenes* infection in children who present with pulmonary, neurological, osteoarticular or skin infections, or even severe cases of septic shock. Since there are no symptoms that could suggest the presence of the bacteria and help on differential diagnosis, epidemiological data can be useful for raising awareness of the possibility of this infection.

## Data Availability

The database that originated the article is available with the corresponding author. CAAE: 78366024.4.0000.5119
